# Acute pre-operative ibuprofen improves cognition in a rat model for postoperative cognitive dysfunction

**DOI:** 10.1186/s12974-021-02206-y

**Published:** 2021-07-08

**Authors:** Klaske Oberman, Iris Hovens, Jacco de Haan, Joana Falcao-Salles, Barbara van Leeuwen, Regien Schoemaker

**Affiliations:** 1 Department of Neurobiology, GELIFES, University of Groningen, Nijenborgh 7, 9747 AG Groningen, The Netherlands; 2 Department of Medical Oncology, University Medical Center Groningen, Groningen, The Netherlands; 3 Department of Microbial Ecology, GELIFES, University of Groningen, Groningen, The Netherlands; 4 Department of Surgery, University Medical Center Groningen, Groningen, The Netherlands

**Keywords:** Cognition, Inflammation, Neuroinflammation, Ibuprofen, Postoperative cognitive dysfunction

## Abstract

**Background:**

Inflammation is considered a key factor in the development of postoperative cognitive dysfunction (POCD). Therefore, we hypothesized that pre-operative anti-inflammatory treatment with ibuprofen would inhibit POCD in our rat-model.

**Methods:**

Male Wistar rats of 3 or 23 months old received a single injection of ibuprofen (15 mg/kg i.p.) or were control handled before abdominal surgery. Timed blood and fecal samples were collected for analyses of inflammation markers and gut microbiome changes. Behavioral testing was performed from 9 to 14 days after surgery, in the open field, novel object- and novel location-recognition tests and Morris water maze. Neuroinflammation and neurogenesis were assessed by immune histochemistry after sacrifice on postoperative day 14.

**Results:**

Ibuprofen improved short-term spatial memory in the novel location recognition test, and increased hippocampal neurogenesis. However, these effects were associated with increased hippocampal microglia activity. Whereas plasma cytokine levels (IL1-β, IL6, IL10, and TNFα) were not significantly affected, VEGF levels increased and IFABP levels decreased after ibuprofen. Long-term memory in the Morris water maze was not significantly improved by ibuprofen. The gut microbiome was neither significantly affected by surgery nor by ibuprofen treatment. In general, effects in aged rats appeared similar to those in young rats, though less pronounced.

**Conclusion:**

A single injection of ibuprofen before surgery improved hippocampus-associated short-term memory after surgery and increased neurogenesis. However, this favorable outcome seemed not attributable to inhibition of (neuro)inflammation. Potential contributions of intestinal and blood-brain barrier integrity need further investigation. Although less pronounced compared to young rats, effects in aged rats indicate that even elderly individuals could benefit from ibuprofen treatment.

## Background

Surgery may evoke an impairment of cognitive functions, including loss of memory, information processing, attention, and cognitive flexibility, which can persist for months to years [1]. This postoperative cognitive dysfunction (POCD) is mostly seen in elderly patients, affecting quality of life and dependency in daily living [2–4].

POCD has been associated with postoperative inflammation, neuroinflammation, and neuronal dysfunction [1, 5, 6]. The inflammatory response evoked by the surgical trauma may become derailed, and is reflected in the brain as neuroinflammation by diffusion or transport of inflammatory factors over the blood-brain barrier (BBB) or stimulation of autonomic nerve fibers [7–9]. Therefore, interfering with the surgery-induced inflammatory response may prevent POCD. Indeed, central blockade of pro-inflammatory cytokines such as TNF-α, IL-1β, and IL-6, as well as treatment with the anti-inflammatory broad spectrum antibiotic minocycline have been shown to attenuate POCD development [10–13]. However, inhibition of peripheral inflammation may not be enough to recover cognitive impairment, as shown in a model of POCD after bile duct ligation [14]. Gut microbiome changes [15], increased intestinal permeability [16], activation of the gut-brain axis [17], and loss of blood-brain barrier (BBB) integrity [14] may collectively contribute to (neuro)inflammation and POCD.

Ibuprofen is widely used as anti-inflammatory medication [18] and may represent general therapeutic potential of non-steroidal anti-inflammatory drugs (NSAIDs) in POCD. Ibuprofen was shown to inhibit lipopolysaccharide-induced cognitive dysfunction and neuroinflammation in rats [19]. Chronic ibuprofen treatment improved cognition after abdominal surgery in mice [20]. Moreover, ibuprofen treatment was associated with distinct microbial profiles [21]. Patients undergoing abdominal surgery showed elevated levels of intestinal fatty acid binding protein (IFABP) levels, as marker for intestinal injury [22]. Intestinal injury can facilitate intestinal leakage/translocation of bacteria or bacterial products. In concert to altered gut microbiota due to surgery [23], this may contribute to the inflammatory response after surgery. Hypoxia-inducible factor-1α and its target gene vascular endothelial growth factor (VEGF) were associated with BBB disruption and consequently cognitive impairment [24]. However, despite complete reversal of post-surgical BBB leakage and hippocampal apoptosis, ibuprofen was not able to inhibit cognitive dysfunction after bile duct ligation [14]. These pieces of evidence suggest additional effects of ibuprofen that may be relevant for inflammation-associated neuroinflammation and POCD.

Over the last decade, we developed a rat model for POCD closely mimicking the clinical setting. In this model, we showed temporal cognitive impairment associated with systemic inflammation and neuroinflammation (combined termed as (neuro)inflammation) in young healthy rats [25] and exaggerated POCD and (neuro)inflammation when known risk factors in patients were superimposed, including aging [26, 27], type of surgery [28], and inflammation history [29].

The aim of the present study was to examine the effects of pre-operative ibuprofen administration on (neuro)inflammation and consequently POCD in our rat model.

## Methods

### Animals and housing

Male outbred Wistar rats (RjHAN:WI) were obtained from Janvier Labs (Saint-Isle, France). Rats were housed in groups of 2-3 individuals and habituated to the animal facility for at least 3 weeks before entering the experimental protocol. Rats at 3 months (young) or 23 months of age (aged) were kept in climate-controlled animal rooms (temperature 20 ± 2 °C, humidity of 50 ± 10%), at 12:12 reversed light:dark cycle. Food and tap water were available ad libitum. All experiments were approved by the local animal experiment and welfare committee (Dier Experiment Commissie, Groningen, The Netherlands).

### Design

In our previous studies, we established the rat model for POCD [25–27, 29], closely mimicking the surgical procedures in the hospital. Since we repeatedly and reproducibly showed the effects of surgery in young and aged rats in these studies, in order to limit the number of animals in the present study, we did not include non-surgical controls but focused on potential effects of treatment.

**Fig. 1:**
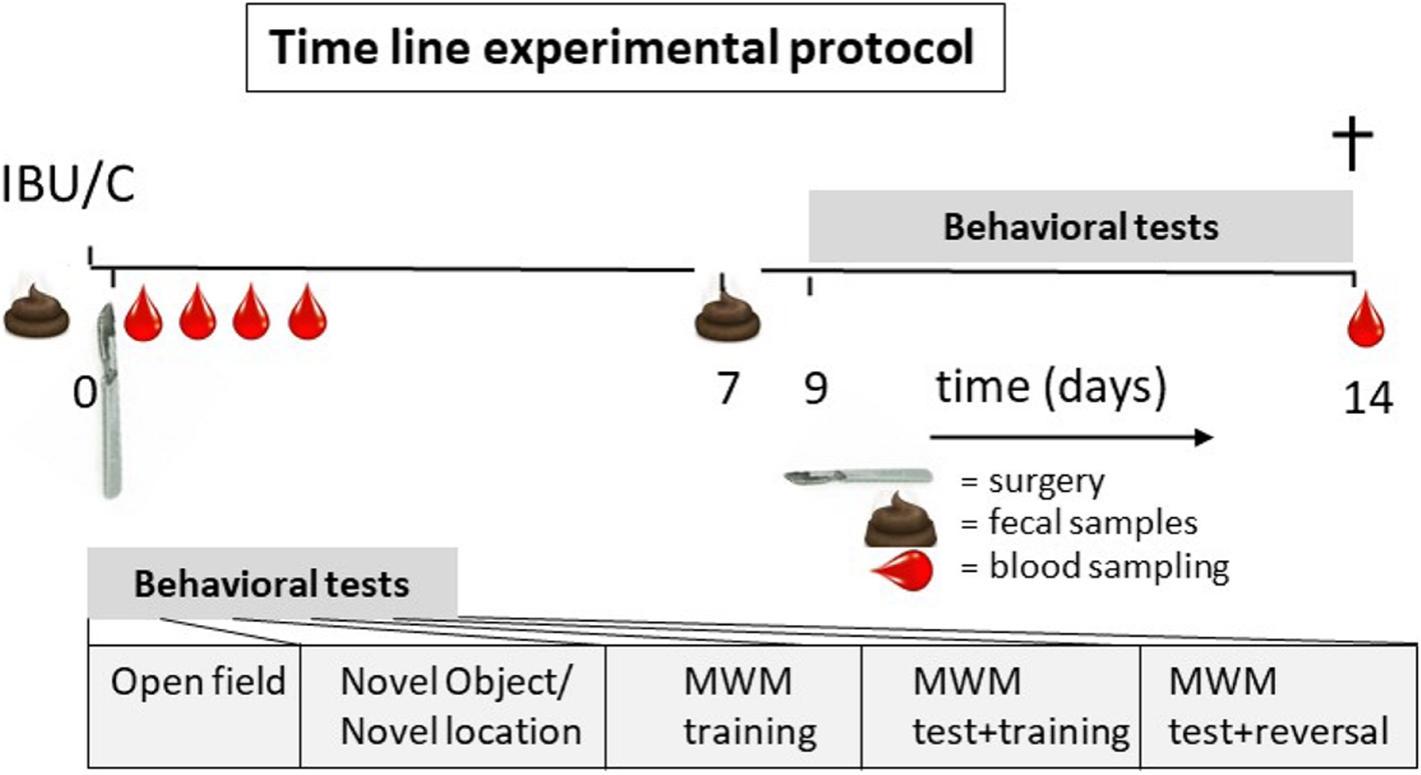
Time line of the experimental protocol. Ibuprofen (IBU)-treated rats (young *n* = 12; aged *n* = 11) received a single i.p. injection of ibuprofen 30 min before surgery; control (C) rats (young *n* = 14; aged *n* = 11) were handled. Rats were sacrificed (+) at day 14. Blood samples were collected at 30 min, and 1, 6, and 24 h after surgery and at sacrifice. Fecal samples were collected before and 7 days after surgery. MWM, Morris water maze

All rats were habituated to repeated handling in the week prior to the experiment. On the day of surgery, rats were randomly assigned to one of the experimental groups, and entered the protocol according to the schedule in Fig. 1. Rats were fasted from 2 h before lights off and housed individually 2 h before surgery. During the latter 2 h, baseline fecal samples were collected. Young and aged rats in the ibuprofen groups (IBU, *n* = 23) received an i.p. injection of ibuprofen (15 mg/kg in sterile saline; 1 ml/kg) 30 min before surgery. Rats in the control groups were shortly handled, but received no pre-operative intervention (C, *n* = 25). Because a well-regulated inflammatory response is essential for effective wound healing, the present study aimed at inhibition of inflammation during the surgical procedure by pre-operative administration, rather than extended ibuprofen treatment.

Rats were subjected to abdominal surgery and were equipped with a permanent jugular vein catheter for timed blood sampling. Fecal samples were collected 7 days after surgery, to study potential sequential and long-term changes in the microbiome to be associated with the subsequent behavioral test outcomes. Between postoperative day 9 and 14, rats were subjected to behavioral testing. Exploratory and affective behaviors were assessed in the open field (OF). Short-term object and spatial memory were assessed by the novel object recognition (NOR) and novel location recognition (NLR) tests. Long-term spatial learning, spatial memory, and cognitive flexibility were tested in the Morris water maze (MWM). Brains were collected on postoperative day 14 to determine microglial activity and the number of young maturing neurons (neurogenesis).

### Surgery

The surgical intervention was performed as previously described [25]. Briefly, rats were anesthetized with sevoflurane (3% sevoflurane, 30% oxygen, 0.35 L/min) and buprenorphine analgesia (3 μg/kg s.c., at the start of surgery). Next, the intestines were exteriorized and the upper mesenteric artery was clamped for 30 min, to mimic this aspect of the procedure in abdominal surgery in humans (BL van Leeuwen). Within these 30 min, a permanent indwelling jugular vein catheter was placed, for one to match the insertion of a permanent venous line in patients, and secondly to facilitate blood sampling without repeated anesthesia.

### Behavior

Behavioral testing was performed in the first half of the dark phase under dim light conditions, in a room adjacent to the housing room, as previously described in detail [25]. The OF test was performed on postoperative day 9 to assess anxiety and exploratory behavior, using time spent in the center of the arena and the total distance moved as outcome measures, respectively. More time in the center indicated less anxiety, while more distance moved was interpreted as more exploratory behavior (more interest in environment).

The NOR and NLR tests were performed on postoperative day 10. Object and location preferences were expressed by the time spent exploring the novel or relocated object as percentage of the total time spent on object exploration. These were used as measure for object and location recognition, respectively. Results of rats that did not explore the objects (exploration time less than 3%) were omitted from the statistical analysis.

MWM testing was performed on days 11-13 after surgery. The average escape latency to the platform of each training session (5 training sessions with 3 entries each) was used to construct a spatial learning curve. The area under the curve (AUC) was used as a measure for spatial learning performance.

Long-term memory was obtained in a probe trial. For that, the platform was removed from the water maze, and the rats were allowed to search for the platform. Total distance moved was used as measure for active searching, while time spent in the target quadrant (TQ, the quadrant with the previous location of the platform) and the number of times rats swam over the previous position of the platform was determined as measure for long-term spatial memory. In addition, to the regular probe trial at the third day (24 h after training session 5), an extra probe trial was performed on the second day (24 h after training session 3), to obtain information about the development of long-term memory. Pilot studies indicated that this procedure did not disturb spatial learning.

To determine cognitive flexibility, reversal training (2 trainings sessions, with 3 entries each) was performed on the third day of the protocol. For that, the platform was relocated to the quadrant opposing the original TQ. The AUC of the average escape latency to the platform was used as a measure for cognitive flexibility.

### Blood sampling and ELISA assays

Blood was collected via the jugular vein catheter at 30 min, 1 h, 6 h, and 24 h after clamp removal. Blood was immediately transferred to sampling tubes containing 20 μl/ml saturated EDTA solution, stored on ice, and centrifuged for 10 min at 2600g at 4 °C. Plasma was collected and stored at −80 °C. Plasma concentrations of tumor necrosis factor-α (TNFα), interleukins (IL) 1β, 6 and 10, and vascular endothelial growth factor (VEGF), were determined using the Bio-Plex Pro Rat 5-plex cytokine assay (Bio-Rad Laboratories BV, Veenendaal, The Netherlands). HIF-1α/VEGF signaling seems to be the upstream mechanism of isoflurane-induced cognitive impairment, and provides a potential target for POCD, as measure for BBB disruption [24]. Additionally, plasma levels of intestinal fatty acid binding protein (IFABP) were determined as measure for loss of intestinal integrity and inflammation [30], using a rat I-FABP ELISA kit (R&D systems Inc., Minneapolis, USA) following the manufacturer’s instructions. Since the hippocampus is most sensitive to neuroinflammation [25], and IL1-β was indicated to play a major role in the development of POCD [11, 31], IL1-β levels were measured in hippocampal tissue. Hippocampi, collected at sacrifice, were homogenized by sonification. The supernatant was collected, and further processed for ELISA [25] to measure IL1-β levels, according to manufacturer’s instructions (rat IL1-β ELISA kit, Invitrogen, Vienna, Austria). The hippocampal IL1-β levels were expressed per milligram of hippocampal tissue.

### Immunohistochemistry

At day 14, rats were sacrificed. Under deep pentobarbital anesthesia, rats were transcardially perfused with cold saline and brains were harvested. From half of each brain, the hippocampus was dissected, immediately frozen in liquid nitrogen and stored at −80 °C for molecular analysis (see above). The other half brain was emersion fixed in 4% PFA for 2-3 days, washed in 0.01M PBS, and dehydrated using a 30% sucrose solution. Tissues were quickly frozen in liquid nitrogen and stored at −80 °C until 25 μm coronal sections were cut using a microtome.

To visualize microglial cells and young maturing neurons, sections were stained for ionized-binding adaptor protein (IBA)-1 and doublecortin X (DCX), respectively, as previously described in detail [25, 32]. To analyze IBA-1 stained sections, images were taken at 200× magnification of the hippocampal cornu ammonis (CA)1, CA3, dentate gyrus inner blade (DGib), and hilus of 3 sections per rat per area, and analyzed blinded for previous experimental procedures. The number of microglia, coverage, and average cell body size were determined with the image-pro plus software (Image Pro Plus 6.0, Media Cybernetic Inc. Rockville, USA). Neuroinflammation was expressed as cell body area/total cell area, as morphological measure for microglia activity [32].

Neurogenesis was obtained by measuring DCX positive cells in the dentate gyrus (DG) of the hippocampus. Images of DCX stained sections (3 per rat), were taken at 50× magnification of the DG. The number of labeled neuronal cell bodies was counted manually by two independent researchers blinded for the experimental groups, and corrected for the length of the DG (mm).

### Microbiome extraction and analyzes from fecal samples

Fecal samples were collected from spontaneous releases within 2 h after individual housing at baseline (before surgery) and on postoperative day 7 (Fig. 1). Samples were stored at −20 °C until further analysis. DNA was extracted using the MoBio PowerFecal DNA Isolation Kit (MoBio Laboratories, Carlsbad, CA) according to kit protocol, except for the use of a bead beater (mini-bead beater, BioSpec Products, Bartlesville, OK) for three cycles of 60 s [33]. DNA samples were stored at −20 °C until further use. For microbiome analyzes, the bacterial 16S ribosomal RNA (rRNA) gene (V4-V5 region) was amplified in 25 μl PCR reaction in triplicates. Amplicons of each sample were pooled and run on a 1% agarose gel (w/v). Bands containing the correct size were excised and purified using the QIAEX II Gel Extraction Kit (QIAGEN, Hilden, Germany). Purified amplicons were quantified using a NanoDrop 2000 (Thermo Scientific, Waltham, MA). Samples were sequenced at GENEWIZ (South Plainfield, NJ) on an Illumina MiSeq platform in a 2 × 300bp paired-end (PE) configuration and analyzed using the Quantitative Insights Into Microbial Ecology (QIIME) [34]. Obtained sequences were trimmed for quality and then binned into operational taxonomic units (OTUs) at 97% nucleotide identity using UCLUST. A representative sequence for each OTU was aligned against the Greengenes coreset using PyNAST. The QIIME toolkit was also used to calculate the weighted and unweighted UniFrac distance matrices for community comparisons, as well as α-diversity measurements, including bacterial richness, based on the number of species per sample (number of observed OTUs) and bacterial diversity (Shannon *H*’), considering both the number of species as well as their frequencies (equitability). The overall microbial communities were compared (β-diversity) using principal coordinate analysis, which provide indication of the distribution of the taxa and relative abundance of detected species among age and treatment.

### Statistical analysis

Data are reported as mean ± SEM. Statistical analysis was performed using SPSS (IBM SPSS Statistics, Version 22, Armonk, NY). Data that exceeded mean ± twice standard deviation of its group are regarded as outliers, and were omitted (maximally 1 left out per group). Effects were analyzed using two-way ANOVA, with age (young or aged) and intervention (ibuprofen or control treatment) as independent variables. When significant effects were present, one-way ANOVA and post hoc LSD analysis were used to compare groups. Effects were regarded statistically significant when p ≤ 0.05.

For microbiome analyses, we assessed β-diversity (differences between community composition) between different age and treatment groups, using permutational multivariate analysis of variance (PerMANOVA Primer 6 version 6.1.16 & PERMANOVA+ version 1.0.6). Changes in β-diversity were calculated as the difference, in percentage, between the average values per time point within treatment based on the weighted UniFrac distance.

Associations between parameters were analyzed using Spearman correlations. Since many parameters showed age-associated differences, regression analyses were performed per age group. Correlations with *p* values of ≤ 0.05 were considered statistically significant.

## Results

### General

Surgery did not cause mortality in young or aged rats. Maximum body weight loss after surgery was significantly higher in aged compared to young rats (young C, 5.0 ± 0.4%, *n* = 14; young IBU, 4.9 ± 0.7%, *n* = 11; aged C, 8.2 ± 0.6%, *n* = 11; aged IBU, 7.1 ± 1.3%, *n* = 9; F_3,44_ = 25.20, *p* < 0.001), but no intervention (ibuprofen) (F_3,44_ = 0.49, *p* = 0.489) nor age*intervention interaction effects (F_3,44_ = 0.02, *p* = 0.888) were observed.

### Postoperative behavior

**Fig. 2:**
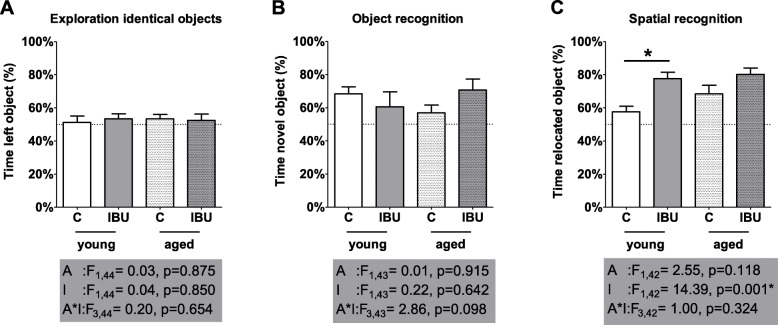
Preference for the objects in the setting with 2 identical objects (side preference; A), for the novel object in the novel object recognition test (B) and for the relocated object in novel location recognition test (C) (mean ± SEM). Dotted line, reference line (50%) for random exploration. C, control (young *n* = 13; old *n* = 11); IBU, ibuprofen treatment (young *n* = 10-11; old *n* = 8-9). F, F statistics for A, main effect of age; I, main effect of intervention; A*I, interaction effect of age and intervention. *p < 0.05

Figure 2 displays short-term object and spatial memory, as obtained from the NOR and NLR test. One rat (young IBU) was excluded from analysis of the NOR, and two rats (young IBU and aged C) were excluded from analysis of the NLR, because they spent less than 3% of the time on exploration of the objects. None of the groups showed side preference when presented with two identical objects in the exploration phase (Fig. 2A). There was no effect of age, intervention or age*intervention in the NOR (Fig. 2B). However, pre-operative ibuprofen administration improved the new location recognition in the NLR; post hoc tests revealed a statistically significant effect in young (*p* = 0.001) and a strong tendency toward improvement in aged rats (*p* = 0.063) (Fig. 2C).

**Fig. 3:**
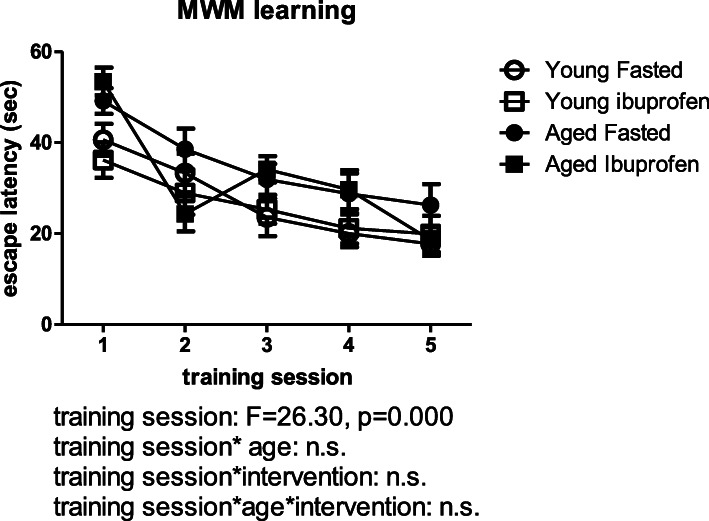
Learning curve as latency to find the platform in the Morris water maze (MWM) for the different experimental groups. C, control (young *n* = 13; old *n* = 12); IBU, ibuprofen treatment (young *n* = 11; old *n* = 9). F, F statistics for A, main effect of age; I, main effect of intervention; A*I, interaction effect of age and intervention

Spatial learning is presented in Fig. 3. This figure shows an overall slightly better learning curve for young versus aged rats, but no effect of ibuprofen treatment. Moreover, no disturbance of the additional probe trial (between training session 3 and 4) on the learning curve was observed. Table 1 shows the other behavioral parameters that were measured in the OF, NOR, NLR, and the MWM. While age significantly affected almost all parameters, ibuprofen administration only increased the distance moved during the MWM probe trials. No significant age*intervention effects were observed.

**Table 1:** Analyzed behavioral parameters in the open field, NOR, NLR, and MWM (mean ± SEM). Open field behavior was characterized by total distance moved (distance) and the percentage of time in the center (time center). Total exploration of objects in phase 1 (2 similar objects), 2 (NOR), and 3 (NLR) of the NOR and NLR was determined. In the MWM, the area under the curve (AUC) for the training sessions (learning) and reversal training sessions (reversal), the time in the target quadrant (TQ) during the first (test 1) and second probe trial (test 2) and the distance moved (distance) were determined. *C* control, *IBU* ibuprofen. F statistics (F) are displayed for age (A), intervention (I), and age*intervention (A*I) effects. * *p* < 0.05

	Young		Aged		F-statistic		
	C	IBU	C	IBU	A	I	A*I
**Open field**							
Distance (cm)	3192 ± 115	3399 ± 80	2299 ± 124	2253 ± 129	78.6*	0.48	1.20
Time center (%)	17 ± 3	17 ± 3	8 ± 2	7 ± 1	19.7*	0.18	0.09
**NOR/NLR**							
Object exploration 1 (%)	27 ± 1	22 ± 2	23 ± 2	20 ± 2	1.62	3.91	0.23
Object exploration 2 (%)	16 ± 2	14 ± 2	10 ± 1	8 ± 3	8.35*	0.82	0.00
Object exploration 3 (%)	16 ± 2	12 ± 2	10 ± 2	10 ± 2	4.36*	1.14	1.55
**MWM**							
Learning (AUC)	107 ± 10	104 ± 7	137 ± 9	124 ± 8	8.14*	0.80	0.31
Reversal (AUC)	21 ± 2	25 ± 2	33 ± 3	32 ± 4	12.3*	0.34	0.54
TQ Test 1 (%)	25 ± 2	23 ± 1	32 ± 2	27 ± 2	7.22*	3.09	0.91
TQ Test 2 (%)	30 ± 2	34 ± 4	31 ± 2	29 ± 2	0.63	0.15	0.87
Platform crossings test 1	1.9 ± 0.5	2.1 ± 0.2	2.8 ± 0.4	2.4 ± 0.5	1.75	0.02	0.60
Platform crossings test 2	2.8 ± 0.2	3.4 ± 0.4	2.2 ± 0.4	2.3 ± 0.5	5.34*	1.16	0.37
Distance test 1 (cm)	1144 ± 50	1256 ± 50	947 ± 70	1081 ± 47	10.4*	4.57*	0.03
Distance test 2 (cm)	1105 ± 51	1203 ± 41	898 ± 75	1041 ± 50	10.2*	4.32*	0.15

### Microglia activity

Microglial activity in the prefrontal cortex (PFC), striatum (STR), and hypothalamus (HYP) were increased with age, but not significantly affected by ibuprofen treatment (Table 2). However, in neither area ibuprofen treatment showed a tendency to reduce microglia activity.

**Table 2:** Microglia activity, measured as cell body area/total cell area (%) in the different brain areas. *C* control, *IBU* ibuprofen. F statistics (F) are displayed for age (A), intervention (I), and age*intervention (A*I) effects. *p < 0.05

	Young		Aged		F-statistic		
n	C	IBU	C	IBU	A	I	A*I
	12	8	9	6			
**Brain area**							
Prefrontal cortex	15.5 ± 1.2	15.8 ± 1.1	18.7 ± 0.8	20.5 ± 1.2	15.2*	ns	ns
Striatum	12.0 ± 1.0	12.1 ± 0.6	15.6 ± 0.9	16.6 ± 0.9	18.5*	ns	ns
Hypothalamus	13.1 ± 1.0	13.5 ± 1.6	18.0 ± 1.7	28.5 ± 0.9	12.6*	ns	ns

**Fig. 4:**
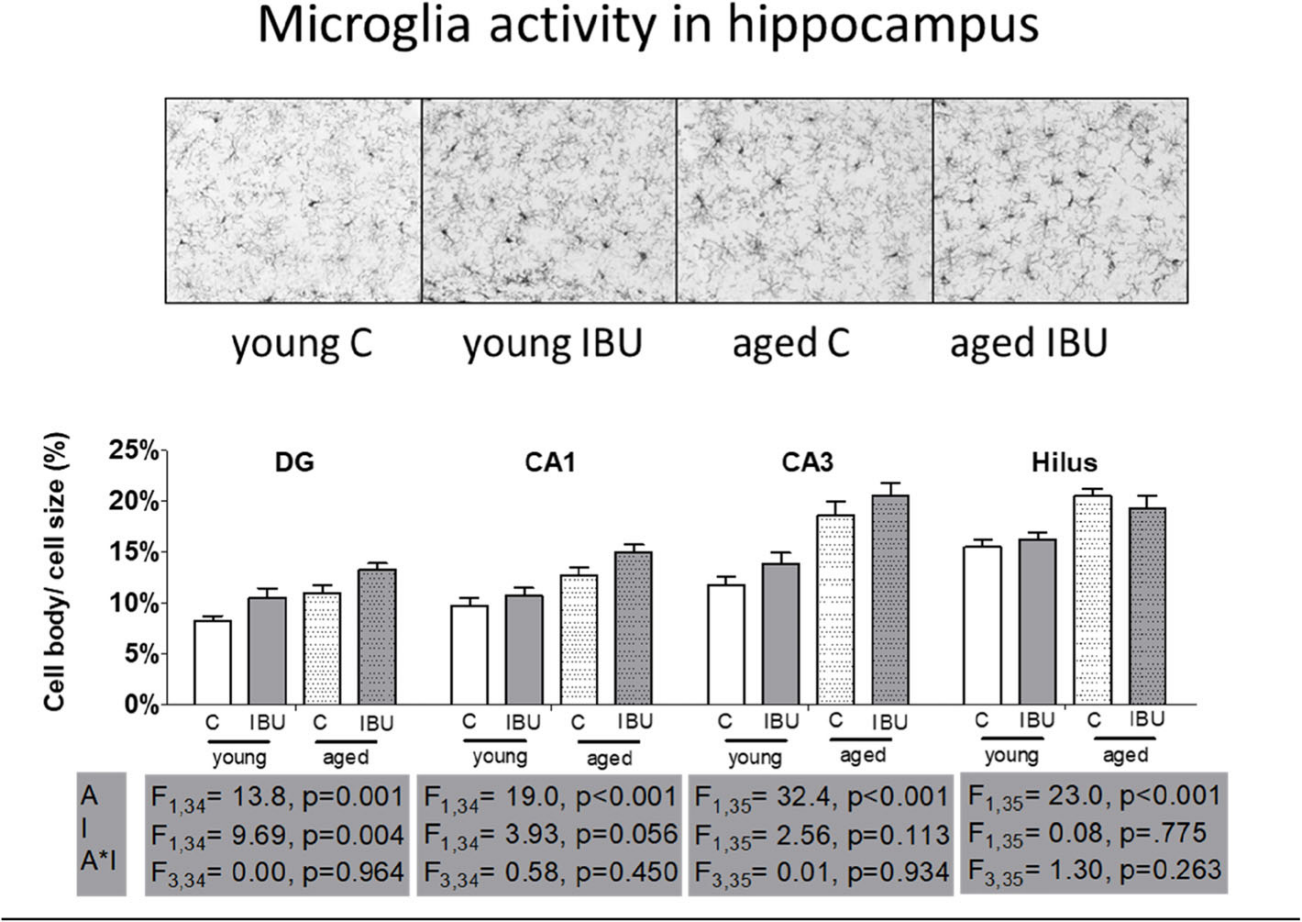
Microglial activity in hippocampal areas (mean ± SEM). Top, representative images of microglia staining of the dentate gyrus. Bottom, ratio between the cell body size and total cell size in the hippocampal regions dentate gyrus inner blade (DG), cornu ammonis 1 (CA1), cornu ammonis 3 (CA3), and Hilus. C, control (young *n* = 12; old *n* = 9-10); IBU, ibuprofen (young *n* = 8; old *n* = 6). F statistics (F) are displayed for age A, main effect of age; I, main effect of intervention; A*I, interaction effect of age and intervention

Figure 4 displays microglia activity in the different hippocampal areas. There was a significant effect of age on microglia activity in all areas. Surprisingly, ibuprofen treatment increased microglial activity in the dentate gyrus, and a similar trend was observed in the CA1 area. There were no significant age*intervention effects. Moreover, in none of the hippocampal areas, ibuprofen-treated rats displayed lower microglia activity than their age-matched controls. In young, but not in aged rats, microglia activity in the hippocampus was positively correlated with time in the center area of the open field (*r* = 0.52; *p* = 0.018) and long-term spatial memory, measured as time in target quadrant in the MWM (*r* = 0.47; *p* = 0.036). No correlations with NOR and NLR test outcomes were seen.

### Neurogenesis

**Fig. 5:**
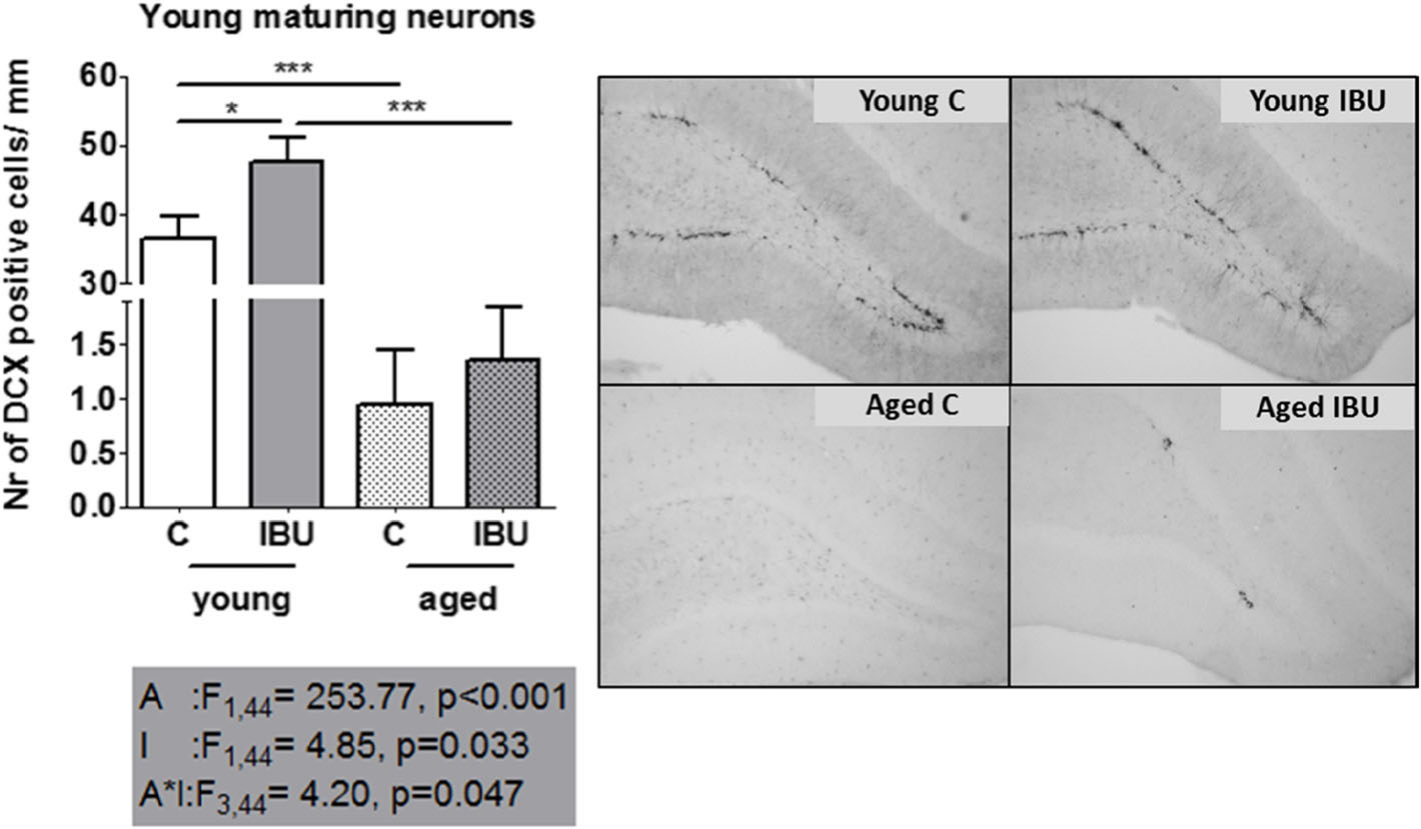
Neurogenesis measured as DCX positive cells (mean ± SEM). Left, the number of DCX positive cells in the dentate gyrus (DG) corrected for the length of the DG in mm (cells/mm). Right, representative images of DCX stained sections of the dentate gyrus. C, control (young *n* = 13; old *n* = 11), IBU, ibuprofen (young *n* = 11; old *n* = 9). F statistics are displayed for: A, main effect of age; I, main effect of intervention; A*I, interaction effect of age and intervention. *p < 0.05, ***p < 0.001

The number of young maturing neurons in the hippocampal dentate gyrus as a measure for neurogenesis is displayed in Fig. 5. There was a significant effect of age and intervention, as well as a significant age*intervention interaction effect. Ibuprofen treatment increased the number of young maturing neurons, particularly in young rats. Aged animals showed low neurogenesis leaving very little opportunity for rescue by ibuprofen.

In young, but not in aged rats, microglia activity in the hippocampus was positively correlated to neurogenesis (*r* = 0.55, *p* = 0.012). Moreover, neurogenesis was found to be positively correlated to long-term spatial memory, measured by time in target quadrant in the probe trail of the MWM (*r* = 0,43; *p* = 0.035).

### Inflammatory markers

**Fig. 6:**
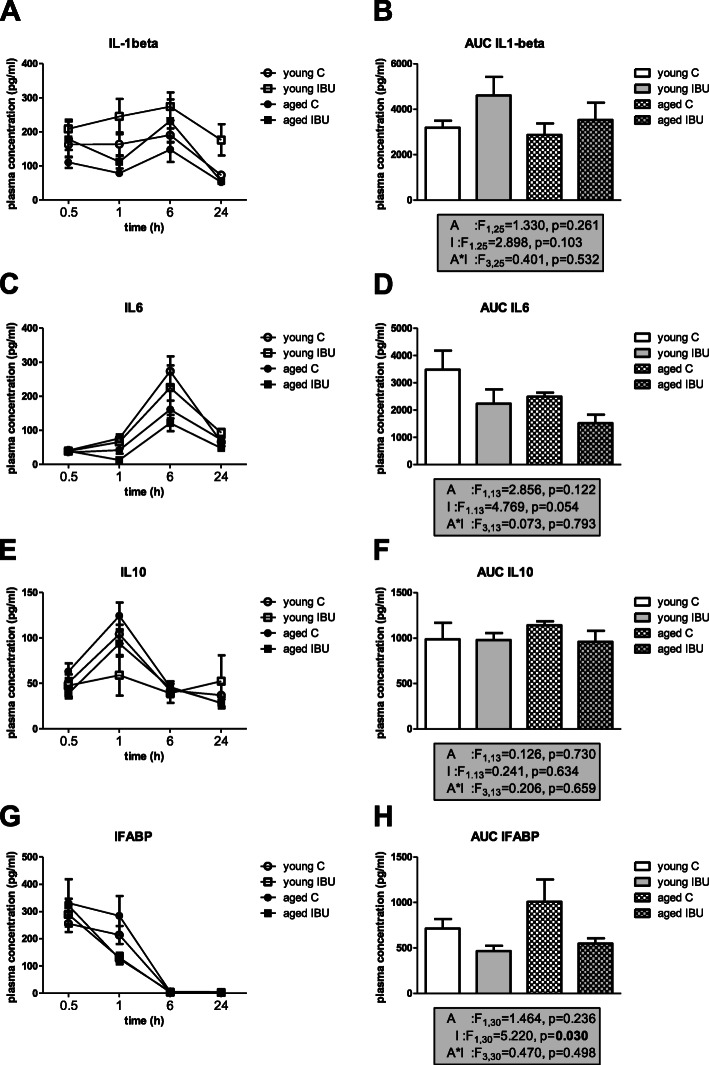
Time course for plasma markers (A, C, E, G) and area under the curve (AUC; B, D, F, H) during the first 24 h after surgery in the different experimental groups. C, control; IBU, ibuprofen; IFABP, intestinal fatty acid binding protein (*n* = 7-12); IL6, interleukine-6 (*n* = 3-4); IL10, interleukin-10 (*n* = 3-6); IL1-β, interleukin-1-β (*n* = 5-8). F statistics are displayed for A, main effect of age; I, main effect of intervention; A*I, interaction effect of age and intervention

**Fig. 7:**
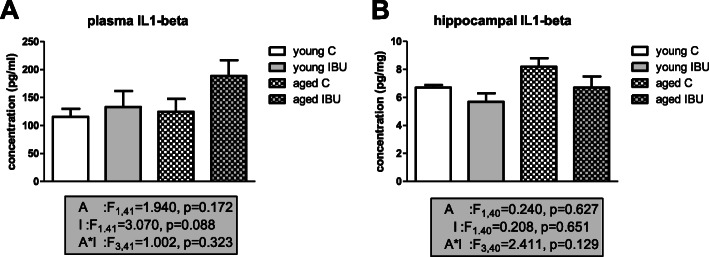
Plasma and hippocampal levels of IL1-β, at the time of sacrifice, 14 days after surgery. C, control (young *n* = 13; old *n* = 10), IBU, ibuprofen (young *n* = 10-11; old *n* = 8-9). F statistics are displayed for age (A), intervention (I), and age*intervention (A*I) effects

To measure effects on systemic inflammation, plasma levels of TNFα, IL1-β, IL6, IL10, VEGF, and IFABP were analyzed. Detectable levels for TNFα and VEGF could only be obtained at 1 h after surgery. Although for these peak TNFα levels, no significant effects of age, ibuprofen, or age*ibuprofen interaction were observed (young control, 115 ± 29 pg/ml, *n* = 9; young IBU, 149 ± 60 pg/ml, *n* = 5; aged control, 132 ± 12pg/ml, *n* = 9; aged IBU, 212 ± 87 pg/ml, *n* = 7), ibuprofen groups tended to have even higher levels than their age-matched controls. For VEGF levels, a significant effect of age (F_1.29_ = 10.436, *p* = 0.003) and a strong tendency for intervention effects (F_1.29_ = 3.97, *p* = 0.057) were seen (young control, 125 ± 38 pg/ml, *n* = 10; young IBU, 260 ± 76 pg/ml, *n* = 7; aged control, 24 ± 5 pg/ml, *n* = 6; aged IBU, 69 ± 17 pg/ml, *n* = 7). After anti-inflammatory treatment with ibuprofen, increased rather than decreased levels were detected. In line with this observation, peak levels of IL1-β (at 6 h) had increased after ibuprofen treatment (*p* = 0.037). The time courses over the first 24 h after surgery (Fig. 6) showed different patterns for the different markers, with IFABP peaking earliest, IL10 in between, and IL6 and IL1-β latest. Early effects were analyzed as the area under the curve (AUC) for the first 24 h after surgery. No age-related effect on the inflammatory response was observed. Ibuprofen only significantly decreased plasma levels of IFABP, while IL6 levels showed a tendency to decline. Levels of IL10 were not affected. IL1-β levels tended to increase early after ibuprofen treatment, an effect still observed at 14 days after surgery (Fig. 7A). Plasma IL1-β levels measured shortly after surgery (AUC first 24 h) significantly correlated to the distance moved in the open field test (*r* = 0.75, *p* = 0.005) in young, but not in aged rats. No further correlations were observed for early or late (sacrifice) plasma IL1-β levels with other behavioral parameters in young or aged rats.

IL1-β levels in the hippocampus were not significantly affected by ibuprofen (Fig. 7B). These hippocampal IL1-β levels negatively correlated to hippocampal microglia activity (*r* = −0.47, *p* = 0.044) and to DCX positive cells (*r* = −0.44, *p* = 0.039) in young, but not in aged rats. Hippocampal IL1-β levels showed no relation with behavior at either age. Moreover, no correlations between hippocampal IL1-β levels and plasma IL1-β levels were observed. In young rats, hippocampal IL1-β, but not plasma IL1-β, significantly correlated with BBB integrity marker VEGF (*r* = −0.62, *p* = 0.014), but not with gut integrity marker IFABP. Moreover, both barrier markers significantly correlated to neurogenesis (DCX positive cells); AUC IFABP, *r* = 0.69, *p* = 0.013; peak VEGF, *r* = 0.70, *p* = 0.003, while peak VEGF levels also correlated with hippocampal microglia activity (*r* = 0.58, *p* = 0.036). AUC IFABP in young rats correlated with distance in the OF (*r* = 0.75, *p* = 0.005). However, neither of these parameters correlated to any cognitive parameter. In contrast, in aged rats AUC I-FABP (*r* = 0.54, *p* = 0.025) as well as peak VEGF (*r* = −0.56, *p* = 0.045) correlated to AUC of spatial learning (MWM).

### Microbiome

**Fig. 8:**
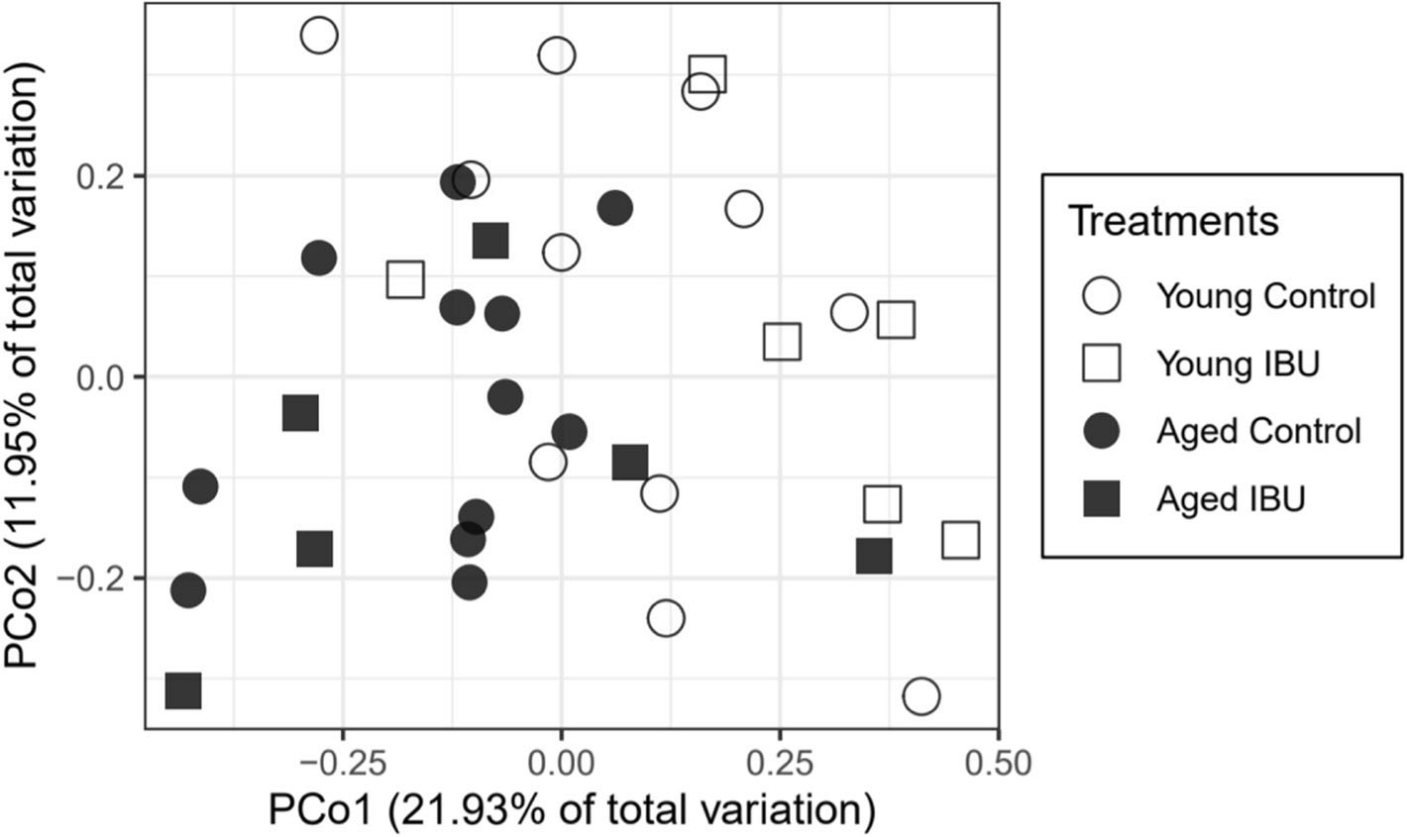
Ordination of the gut microbiome (bacterial communities) based on all samples. The principal coordinate analysis (PCO) plot is based on the weighted UniFrac distance matrix calculated on rarefied OTU abundances (1000 sequences per sample) for all samples. Variance explained by each PCO axis is given in parentheses

The gut microbiome was studied from collected fecal samples that were spontaneously released within 2 h, when rats were individually housed in a clean cage. This resulted in limited number of samples since not all rats had produced samples within this time frame. Changes in the gut microbiome due to surgery, age, and/or intervention were analyzed by performing community comparisons using Principal Coordinate analysis (PCO) of all samples (Fig. 8). The first two coordinates explained 21.9% (horizontal axis) and 12.0% (vertical axis) of the variation, respectively. The graph shows a segregation of microbiome between aged and young rats (PerMANOVA, *p* = 0.001), but no effect of ibuprofen treatment. The latter could be due to the limited number of individuals contributing to the microbiome of the ibuprofen-treated animals. This, however, resulted in a significant effect of ibuprofen-age interaction (*p* = 0.045).

The individual microbiome appeared rather stable as no significant differences between diversity parameters before surgery and 7 days after surgery were observed; α-diversity parameters before surgery highly correlated with values 7 days after surgery (*r* = 0.721; *p* = 0.001). However, whereas the number of OTUs (“species”) per sample was similar in both age groups at baseline (Table 3), at 7 days after surgery, the number of OTUs was significantly higher in aged compared to young rats (effect of age *p* = 0.009). No significant effects of ibuprofen nor of ibuprofen*age interaction were observed. Similarly, the α-diversity parameter that also includes evenness; Shannon H’ index, showed no differences at baseline, but significant effects of age at 7 days (*p* = 0.017), without effect of ibuprofen or interaction. No correlations between these microbiome parameters were found with any of the other measured parameters, neither for young nor for aged rats.

**Table 3:** Number of OTUs (species), Shannon H’ diversity index, and Firmicutes/Bacteroidetes of the gut microbiota in young and old rats before surgery and 7 days after surgery for control (C) and ibuprofen (IBU) treatment. At 7 days after surgery, the number of OTUs as well as Shannon’s index was significantly higher in aged compared to young rats, irrespective of treatment

Treatment	N	Before surgery			7 days after surgery		
		# Species	Shannon’s	Firm/Bact	# Species	Shannon’s	Firm/Bact
Young C	7	579 ± 47	8.4 ± 0.2	0.87 ± 0.11	559 ± 46	8.2 ± 0.3	0.96 ± 0.14
Young IBU	3	529 ± 56	8.1 ± 0.3	0.81 ± 0.16	517 ± 83	8.0 ± 0.5	0.63 ± 0.13
Aged C	6	642 ± 34	8.5 ± 0.3	1.19 ± 0.14	679 ± 15	8.9 ± 0.1	0.95 ± 0.08
Aged IBU	3	632 ± 33	8.5 ± 0.1	0.91 ± 0.12	667 ± 35	8.7 ± 0.2	0.85 ± 0.07

**Fig. 9:**
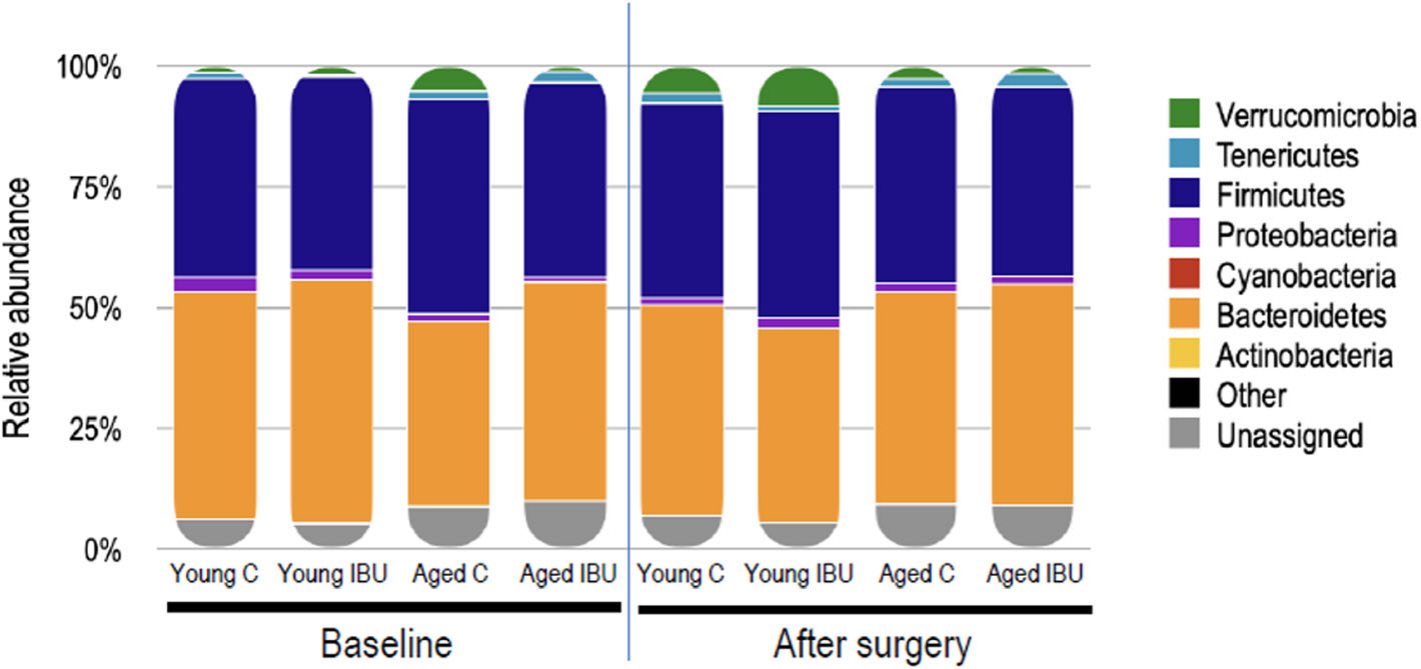
Relative abundance of bacterial phyla, at baseline (before surgery) and 7 days after surgery in young and aged rats. C, control (young *n* = 10; old *n* = 12); IBU, ibuprofen (young *n* = 6; old *n* = 6)

The taxonomic distribution of bacterial species across samples is summarized in Fig. 9. In presurgical samples, a highly significant negative correlation was observed between the relative abundance of Firmicutes and that of Bacteroidetes (*r* = −0.815, *p* < 0.001). In aged rats, the abundance of Bacteroidetes was lower (*p* = 0.037) than in young rats. Ibuprofen consistently lowered the ratio Firmicutes/Barcteroidetes, though not statistically significant (Table 3). Furthermore, surgery in aged rats significantly lowered the abundance of phylum Verrucomicrobia (*p* = 0.014), without effects of ibuprofen. This relative abundance of Verrucomicrobia negatively correlated with species richness and Shannon diversity index (correlations with number of species, *r* = −0.706, *p* = 0.002; with Shannon index *r* = −0.713, *p* = 0.001). Moreover, Verrucomicrobia abundance correlated with time in the center in he OF test (*r* = −0.76; *p* = 0.018). In aged, but not in young rats, relative Verrucomicrobia abundance was negatively correlated to hippocampal IL1-β (*r* = −0.81, *p* = 0.008, *n* = 9).

## Discussion

### General

Surgery-induced (neuro)inflammation may play an important role in the development of POCD. Ibuprofen is widely used as anti-inflammatory medication. Here, the effects of pre-operative ibuprofen on (neuro)inflammation and cognition were investigated in our rat model for POCD. A single injection of ibuprofen 30 min before surgery increased neurogenesis and improved hippocampus-associated cognitive function after surgery. However, this favorable outcome seemed associated with increased, rather than suppressed (neuro)inflammation. Accordingly, elevated VEGF levels may point to increased BBB leakage. Reduced IFABP levels, reflecting gut wall integrity, in concert to the lack of major changes in the gut microbiome might suggest decreased contribution of the gut-brain axis. In general, effects in aged rats appeared similar to those in young rats, though less pronounced. However, for neither parameter, ibuprofen-treated aged rats underperformed compared to control aged rats, suggesting that even a single dose of ibuprofen before surgery may contribute to prevention of POCD in aged individuals. Still, optimal timing/dosing of ibuprofen intervention would require follow-up studies.

### Effect of aging

The major risk factor for developing POCD is an old age. Therefore, in the present study, young as well as aged rats were included. In accordance with our previous studies [26, 27], for most parameters, including open field behavior, learning and memory in the MWM, neuroinflammation and neurogenesis, significant age-related impairment was observed. However, also in agreement with previous studies in young and aged rats [26, 27, 31], systemic markers of inflammation did not show age-related differences. Since IL1-β is suggested to play an important role in POCD [11, 31], this cytokine was measured in hippocampal tissue as well. Similar to 14 days after inflammation induced by E-coli injection [31], hippocampal IL1-β responses did not differ between young and aged rats.

The gut microbiome can contribute to (neuro)inflammation and neurological dysfunction [17]. Moreover, aging [35] as well as (gastrointestinal) surgery, anesthetics, and pre-operative fasting were reported to affect the microbiome [15, 23]. In the present study, gut microbiome diversity parameters indicated age-associated differences, but no effect of surgery. Regarding microbiome composition, higher Firmicutes/Bacteroidetes ratios and higher relative abundance of Verrumomicrobia are associated with cognitive health in aged people [36]. In the present study, the Firmicutes/Bacteroidetes ratio was not affected by age, nor by surgery. The reduced Verrucomicrobia abundance after surgery in aged rats would fit with the impaired cognitive performance after surgery. The negative correlation of Verrucomicrobia abundance with hippocampal IL1-β may support a contribution of the gut-brain axis in (neuro)inflammation in aged rats. Accordingly, the observation that in aged rats, long-term spatial memory correlated with systemic VEGF and IFABP levels, whereas in young rats these correlations were seen with microglia activity and neurogenesis in the brain, may support a contribution of the gut barrier- and BBB functions, and hence the gut brain axis in POCD in aged rats, but not so much in young rats.

### Effects of ibuprofen

#### Cognition

In animal models [14, 19, 20, 37, 38] as well as human studies [39, 40], ibuprofen is successfully shown to reduce systemic as well as neuroinflammation. Since we [25] and others [6] strongly indicated a role for (neuro)inflammation in the development of POCD, we anticipated that ibuprofen would inhibit (neuro)inflammation and improve cognitive performance.

In young rats, hippocampus-dependent memory was shown specifically sensitive to the effects of surgery [11, 12, 25, 39, 40]. Accordingly, ibuprofen improved hippocampus-dependent spatial memory in young rats. In aged rats, however, cognitive impairment extended to hippocampal independent memory, including object recognition and reversal learning [26]. However, ibuprofen treatment did not affect object recognition or reversal learning in aged rats. In contrast, others [20, 37] have shown that ibuprofen following surgery in aged mice could improve both hippocampus dependent and hippocampus independent memory. In addition to the difference in species and test conditions, in those studies, ibuprofen was administered continuously up to 7 [37] or even 14 days [20] after surgery, which may explain the seemingly contrasting findings with our study.

#### (Neuro)inflammation

Although ibuprofen is widely considered an anti-inflammatory drug, in the present study neither of the systemic cytokine levels nor microglia activity in any brain area were found reduced in ibuprofen-treated rats. In line, a POCD study in mice did not reveal effects of ibuprofen on circulating- nor on prefrontal cortex or hippocampus levels of IL1-β, IL6, TNF, or IL10, at 14 days after surgery [20], but ibuprofen did reduce neuroinflammation (microglia and astrocyte activity). In the present study, effects of ibuprofen on hippocampal IL1-β levels and microglia activity appeared to be opposite, supported by the inverse correlation in young rats. Accordingly, whereas hippocampal IL1-β levels negatively correlated to neurogenesis (DCX positive cells), microglia activity correlated positively to neurogenesis. Both microglia activity and neurogenesis positively correlated to long-term spatial memory. Thus, in addition to the unexpected microglia activation after ibuprofen, this observation even appeared associated with the cognitive improvement. Microglia activation in the present study indeed might have contributed to the improved cognitive outcome, but by mechanisms other than neuroinflammation, as microglia can affect cognition in other ways as well, for instance by regulation of synaptic plasticity [41].

Here, most of the effects of ibuprofen observed in young rats appeared similar, but less pronounced, in the aged rats. This may suggest that ibuprofen treatment is less effective in aged rats. Our results corroborate with the literature by showing that the central inflammatory responses to surgery was exacerbated in aged rodents, which could be attributed to a primed immune system [12, 27, 42]. Hence, it is likely that a single bolus of ibuprofen is insufficient to attenuate this primed inflammatory response and, consequently, the detrimental effects on cognitive performance.

Actually, the only statistically significant effects of ibuprofen on circulating markers were observed for IFABP and VEGF. These factors combine inflammation marker properties to reflections of gut permeability [30] and BBB integrity [24], respectively, and may be linked by similar regulatory mechanisms; leaky gut, leaky brain [16]. Increased VEGF levels after ibuprofen are consistent with human endothelial cell culture studies [43], showing intensified lipopolysaccharide-induced VGEF expression and reduced cell survival. However, if VEGF levels indeed reflect BBB integrity [24], then reversal of BBB disruption by ibuprofen in the bile duct ligation POCD study [14] would, in contrast with the present study, suggest ibuprofen-induced reduction of VEGF. However, delayed rather than preoperative ibuprofen treatment hampers comparison between these studies.

IFABP is considered a sensitive marker for intestinal injury, and it was found to be significantly increased after abdominal surgery in patients [22]. Similar to our findings, increased levels in patients return to normal within 24 h after surgery. The significant reduction of IFABP levels after ibuprofen may then reflect protection against intestinal injury and subsequent leakage. Intestinal microbial metabolites are conjectured to affect gut barrier function through the immune system [44, 45] and are now emerging as mediator of cognitive defects. Although specific ibuprofen-induced changes in the microbiome in patients have been described [21], the single ibuprofen administration prior to surgery in our study may be insufficient to show such effects. Lack of microbiome changes, in concert to preservation of gut integrity (reduced IFABP levels), may then suggest reduced contribution of the gut-brain axis after ibuprofen.

#### Timing

Since our hypothesis suggested a rather straight forward process of systemic inflammation-neuroinflammation-neurogenesis-cognitive function, the finding of improved hippocampal associated tasks coinciding not only with higher neurogenesis but also higher hippocampal microglia activity, were rather surprising. However, data from our previous study [25] suggested that time courses for the different aspects do not match perfectly. For instance, persistently reduced neurogenesis after surgery coincided with initially increased microglia activity, followed by normalization, and subsequent declined activity. Spatial memory initially was impaired, but later restored. Hence, interfering with these processes with ibuprofen may induce (different) shifts in the time courses, rather than inhibition of the consecutive aspects indicated in the hypothesis.

In contrast to the single pre-surgical administration of ibuprofen in our study, the referred mice studies [20, 37] continued treatment up to 7 or 14 days after surgery. In concert to this difference in time course of ibuprofen treatment, the time courses of the individual cytokine activations [39] may have played a role. For instance, Huang et al. [20] showed that after surgery circulating Il-6 levels remained elevated up to 72 h, whereas IL1-β levels only appeared elevated at 14 days. Moreover, Huang’s study showed a long-lasting inhibition of systemic inflammation with ibuprofen (reduction of plasma cytokines at 14 days), which may be reflected in lower microglia and astrocyte activation, but not in lower cytokine levels in the PFC and hippocampus. Previously, we showed that plasma IL6 levels peaked at 4-6 h after surgery [25], but brain IL6 was not different at 1, 2, or 3 weeks after surgery. Brain IL1-β showed a biphasic response in the hippocampus; initial increase, followed by a significant decline. In contrast to our present findings, in that study the IL1-β response closely matched with microglia activation [25]. Since the study of Cibelli et al. [11] convincingly underlined the role of IL1-β in the development of POCD by prolonged lowering of IL1-β, the opposite results in the present study, improved POCD at increased circulating IL1-β and neuroinflammation, may indeed relate to differences in time courses. Similar to the present study, a single pre-operative dose of ibuprofen in cholecystectomy patients did not reduce plasma cytokine levels, but was still associated with an improvement of postoperative cognitive outcome [39]. Given the plasma half-life of ibuprofen of less than 2 h, the elevated plasma IL1-β and neuroinflammation in the present study might as well be attributed to a temporal anti-inflammatory effect of ibuprofen, followed by a rebound effect [46].

Finally, delayed ibuprofen treatment, 15-28 days after surgery, was not associated with improved cognition, despite reversal of BBB leakage and inhibition of hippocampal apoptosis [14]. Worth mentioning, POCD studies with an early start of ibuprofen administration leading to cognitive improvement in rats (present study), in mice [20, 37] and in humans [39], may point in favor of early interventions to prevent POCD.

### Limitations

The effects of ibuprofen pretreatment were studied after one dose and at 1 time point. Administered before surgery, ibuprofen treatment was aimed at interference during the surgical procedure, but leaving the subsequent wound healing phase unaffected. Although we are well aware of interfering with processes that are evolving in time (wound healing, (neuro)inflammation, and POCD) [25], by choosing our parameters carefully, clinically relevant data were obtained. Since even this single dose at single time point already showed beneficial effects, it may provide a safe and easy way to inhibit POCD in patients (de Haan and van Leeuwen oral communication).

For microbiome analysis, the collection of spontaneously produced fecal samples within a 2-h period was performed. Unfortunately, this resulted in incomplete sample sets, hampering strong conclusions regarding dynamic changes in the gut microbiome. A sample collection through fecal stimulation should enable the sampling of a larger number of individuals.

Finally, plasma markers were measured with a multiplex system. The advantage of this method is that the levels of more markers could be obtained from the same plasma sample. Disadvantage is that for all markers the same dilution is used. This procedure limits the marker-specific optimalization of dilution, and hence resulted in lack of values that did not meet the detection level of the assay.

## Conclusion

In conclusion, a single dose of ibuprofen preceding abdominal surgery in rats increases hippocampal neurogenesis and improved spatial memory after surgery. However, these favorable effects seemed not directly attributable to the anticipated anti-inflammatory effect of ibuprofen. Further studies are necessary to elucidate on the underlying mechanism, the difference regarding aging, as well as optimize dosing and timing of interventions.

## Data Availability

Data and material are available upon request.

## Abbreviations

AUC: Area under the curve
BBB: Blood-brain barrier
CA: Hippocampal cornu ammonis
DCX: Doublecortin X
DG: Dentate gyrus
HIF: Hypoxia-inducible factor
IBA: Ionized-binding adaptor protein
IBU: Ibuprofen-treated rats
IFABP: Intestinal fatty acid binding protein
IL: Interleukin
NLR: Novel location recognition
NOR: Novel object recognition
NSAID: Non-steroid anti-inflammatory drug
MWM: Morris water maze
OF: Open field
PCO: Principal coordinate analysis
POCD: Postoperative cognitive dysfunction
TNF: Tumor necrosis factor
TQ: Target quadrant
VEGF: Vascular endothelial growth factor
